# Application of telerehabilitation in home care for older adult patients with postoperative hip fractures: A scoping review

**DOI:** 10.1371/journal.pone.0342110

**Published:** 2026-02-12

**Authors:** Chanli Yang, Yingping Fu, Di Du, Xiaojuan Li, Qin Zhou, Yuan Yang, Tianxian Luo

**Affiliations:** 1 School of Nursing, Yunnan University of Chinese Medicine, Kunming, Yunnan, China; 2 Department of Orthopaedics, 920th Hospital of Joint Logistics Support Force, Kunming, Yunnan, China; University of Chile: Universidad de Chile, CHILE

## Abstract

**Background:**

As the older adults population grows, the incidence of hip fractures continues to rise, presenting a major challenge to healthcare systems. Traditional postoperative rehabilitation often struggles with continuity and accessibility, particularly for patients in remote areas. Telerehabilitation, which leverages digital technologies for remote care, is emerging as a potential solution to overcome these limitations and provide more efficient, accessible rehabilitation for older adult patients recovering from hip fractures.

**Objective:**

To conduct a scoping review of studies on the application of telerehabilitation in home care for older adults postoperative hip fractures, aiming to evaluate its effectiveness, methods, and potential for standardization in clinical practice.

**Methods:**

Based on scoping review guidelines, a systematic search was conducted on CNKI, Wanfang Database, CQVIP, CBM, PubMed, Web of Science, Cochrane Library, CINAHL, and Embase, up to August 31, 2025..The included literature was summarized and analyzed.

**Results:**

A total of 18 studies were included. Among these, mobile applications, WeChat platforms, and video interaction systems were the primary methods for home-based care of elderly patients after hip fracture surgery, all utilizing telerehabilitation delivered through video, text, and image-based interventions. A meta-analysis of key outcome measures revealed significant improvements in the telerehabilitation group compared to the control group across several domains: hip function (HHS, P < 0.001), walking ability (6MWT, P < 0.0001), and quality of life (SF-36, P < 0.001). Furthermore, advantages were noted in pain relief (NPRS, P < 0.05) and a reduction in depressive symptoms (HADS-D, P = 0.003). Notably, multiple studies consistently reported significantly higher exercise adherence in the telerehabilitation group compared to the control group (P < 0.05).

**Conclusion:**

Telerehabilitation effectively enhances functional recovery and adherence in older adults after hip fracture surgery. Its success depends on matching interventions to patients’ digital literacy. Future implementation requires standardized protocols and outcome measures to be integrated into professional follow-up care, thereby overcoming existing barriers and maximizing scalability.

**Trial registration:**

OSF Registration DOI: https://doi.org/10.17605/OSF.IO/QYUJM

## Introduction

Hip fracture is a highly prevalent orthopedic disease in the older adult population, especially in people over 65 years of age, often leading to loss of function and high mortality. In China, with the growth of the older adult population, the incidence of hip fractures continues to rise, which has a serious impact on patients’ mobility and quality of life [1]. Falls are the main causative factor for hip fractures, accounting for 89.2% of the incidence. Older adults have a significantly increased risk of falling due to decreased muscle strength and weakened reaction ability, which substantially increases the probability of hip fracture [2]. Currently, total hip arthroplasty (THA) is the mainstay of treatment for hip fractures, but the mortality and complication rates of patients after surgery are still high [3–5]. Postoperative rehabilitation is a key component for patients to regain mobility, prevent re-fracture, and improve their quality of life. However, the traditional home care model, which relies on telephone follow-up, has obvious limitations in terms of service continuity and specialization, and the guarantee of patients’ rehabilitation outcomes is more limited [6,7].

In this context, telerehabilitation has gradually gained attention as an emerging care model. Telerehabilitation is the use of digital technology to establish remote interaction between patients and medical personnel, providing patients with systematic rehabilitation guidance in home or community settings [8]. This model can not only make up for the shortcomings of traditional care, but also provide more convenient rehabilitation services for patients in rural and remote areas or with limited transportation [9]. However, the application of telerehabilitation in older adult postoperative hip fracture patients is still in the exploratory stage, and the current study mainly focuses on individual specific forms or technical means, and the overall application scope and effect have not yet formed a systematic understanding. In order to provide a foundation for the future direction of research and clinical practice, this study, which takes the form of a scoping review, examines the current state of the application of telerehabilitation in the home care of older adults with postoperative hip fractures, summarizes the forms of its application, application tools, and application content, and clarifies the issues of the current research.

## Methods

### Study design

Following the framework of Peters et al.‘ s [10] scoping review, we registered the protocol on the Open Science Framework (OSF: DOI https://doi.org/10.17605/OSF.IO/QYUJM).

### Research questions

What are the forms and contents of the application of remote rehabilitation in the home care of older adults postoperative hip fracture patients?

What is the effect of telerehabilitation on the home care of older adults postoperative hip fracture patients?

What are the problems of the current study?

### Literature search

The system searched the CNKI, Wanfang Database, CQVIP, CBM (Chinese BioMedical Literature Database), PubMed, Web of Science, Cochrane Library, CINAHL (Cumulative Index to Nursing and Allied Health Literature), Embase, and the references of related studies, and the time limit for the search was from the establishment of the database to August 31, 2025. The search was conducted by using both MeSH Terms and free terms. MeSH and free words were jointly used for the literature search. The Chinese search terms were“telerehabilitation/telemedicine/teleconsultation/telemonitoring/telenursing/mobile medical care/mobile health/telehealth/digital health/hip fracture/hip arthroplasty/hip replacement/hip fractures/total hip arthroplasty/continuing nursing care/home nursing care/home Nursing/online nursing/home rehabilitation.” The English search formula is based on PubMed. For example, see Table 1.

**Table 1:** PubMed search strategy.

Step	Search Strategy
#1	(((((((telerehabilitation[MesH Terms]) OR (telerehabilitation[Title/Abstract]) OR (remote monitoring[Title/Abstract]) OR ((Telemedicine[Title/Abstract]) OR (Telemedicine[MeSH Terms]))) OR ((Digital Health[MeSH Terms]) OR (Digital Health[Title/Abstract]))) OR (mobile healthcare[Title/Abstract]))OR (mobile health[Title/Abstract])) OR (Telecare[Title/Abstract])
#2	(((Hip Fractures[MeSH Terms]) OR (Hip Fractures[Title/Abstract])) OR ((Arthroplasty, Replacement, Hip[MeSHTerms]) OR (Arthroplasty, Replacement, Hip[Title/Abstract]))) OR (replacement of total hip[Title/Abstract])
#3	(((((Home Care Services[Title/Abstract]) OR (Home Care Services[MeSH Terms])) OR ((home nursing[MeSH Terms])OR (home nursing[Title/Abstract]))) OR (Online care[Title/Abstract])) OR ((Ambulatory Care Facilities[Title/Abstract])OR(Ambulatory Care Facilities[MeSH Terms]))
#4	#1 AND #2 AND #3

This is the Table 1 legend.

### Inclusion and exclusion criteria of the literature

Inclusion criteria: (1) The study subjects were older adults postoperative hip fracture patients aged ≥60 years, but literature reporting that the mean value of the study subjects was ≥ 60 years and the majority of individuals in the sample met this criterion was accepted; (2) The type of the study was a randomized controlled trial(RCT), a class experiment, an observational study, or a systematic evaluation; (3) The study involved mobile applications(Apps), website platforms, communication software and other forms of telerehabilitation; (4) Have a clear intervention and effect.

Exclusion Criteria: (1) Incomplete article content, unable to access the full text; (2) Repeated publication; (3) Non-Chinese and English.

### Literature screening and data extraction

Duplicate literature was eliminated using Endnote X9 software, and the two researchers then independently performed initial and re-screening. First, two researchers independently screened the titles and abstracts (Y.C.L and F.Y.P), then re-screened them by reading the full text, and finally decided which documents to include. In case of disagreement, it was discussed with the third researcher(D.D). After determining the inclusion of literature, the two researchers(Y.C.L and F.Y.P) independently extracted information on authors, time of publication, country, type of study, sample size, interventions in the test group (form of application, tools of application, content of application), interventions in the control group, and outcome indicators. Finally, members of the research team worked together to summarize and analyze the information.

## Results

### Results of literature screening

A total of 699 pieces of literature were obtained from the search, and 18 pieces were finally included, of which 6 pieces were in Chinese and 12 pieces were in English. The process of literature screening is shown in S1 Fig.

### Basic characteristics of the included literature

This study included 18 articles examining the application of telerehabilitation in home care for elderly patients with hip fractures. Their basic characteristics are detailed in Table 2. Specifically, these studies were conducted across eight countries, primarily in China (n = 8) [9,11–17], followed by the United States (n = 2) [18,19], Spain (n = 2) [20,21], and Italy (n = 2) [22,23]. In terms of study design, the majority were randomized controlled trials (n = 14) [9,11–18,21,23–26], with control groups typically receiving conventional or standard care. The remaining studies were non-randomized controlled trials (n = 4) [19,20,22,27]. Regarding publication timing, the included studies spanned from 2014 to 2025, with most (n = 15) published after 2019.

**Table 2:** Basic characteristics of the included literature (n = 18).

				Telerehabilitation program			
First (year)	Country	Study Design	Sample Size (test group /control group)	Intervention Form and Content	Intervention Frequency/Duration	Control Group	Primary Outcomes
Li Guan et al. (2021)	China	RCT	35/35	Form: WeChat Live Content: Functional exercise, daily activity instruction, dietary instruction	Weekly × 4 weeks	Pamphlet + Telephone	HHS, Patient adherence
Yuanfang Yang etal. (2017)	China	RCT	59/58	Form: Video Interaction Content: Daily living skills, psychological counseling, rehabilitation training	Biweekly home + Weekly remote × 1 month	Routine home rehabilitation	SAS, SDS, SF-36, ADL, etc.
Chunxiang Xu et al. (2019)	China	RCT	50/50	Form: Mobile Platform Content: Functional exercise, health education, real-time interaction	Weekly (Month 1), Biweekly (Months 2–4), Monthly (Month 5–6)	Routine care	HHS, SF-36
Yuhong Niu et al. (2019)	China	RCT	47 (Internet)/ 49 (Telephone)	Form: Mobile App, Telephone Follow-up Content: Telephone Group: complication prevention guidance, health education, rehabilitation training guidance Internet Group: Push the rehabilitation training video and guide the functional exercise online. Support patients to upload photos or videos and real-time feedback	Internet group: Daily × 8 weeks. Telephone group: Weekly (W1-4), Biweekly (W5-8)	No clear control	HHS, BPOMS
Xiumei Tang (2019)	China	RCT	35/35	Form: WeChat Video Content:Functional exercise, real-time feedback	Daily reminders + Biweekly analysis × 6 weeks	Paper-based rehabilitation plan + offline treatment	HOOS, Patient adherence and satisfaction
Qingling Wang et al. (2023)	China	RCT	43/43	Form: Mobile App Content: Rehabilitation videos, goal setting, peer support	5 days/week × 6 weeks	Pamphlet + Telephone	SER, EQ-5D, HADS-A/HADS-D, etc.
Suphawita Pliannuom et al. (2024)	Europe, America, Asia	Systematic reviews and Meta-Analysis	16 studies (1467 participants)	Form: Various Telerehabilitation: telephone (10 studies), web-based software (5 studies), mobile apps (3 studies), sensor monitoring technology (1 study) Content: Education, rehabilitation training, remote monitoring	1-6 months	Routine care	TUG, SPPB, FIM
Mariana Ortiz-Piña et al. (2021)	Spanish	quasi-experimental study	35/36	Form: Web Platform(@ctivehip) Content: Exercise rehabilitation, mobility coaching, weekly video conferencing	3 sessions/week × 12 weeks	Face-to-face rehab	TUG, SPPB, FIM
Lorenzo Lippi et al. (2024)	Italy	prospective cohort study	25	Form: Mobile Monitoring System(Step-App®) Content: Gait assessment, muscle strength assessment, data logging, real-time feedback	Daily + Weekly monitoring × 4 weeks	No control group	6MWT, 10MWT, NRS, etc.
Nancy K. Latham et al. (2014)	USA	RCT	120/112	Form: DVD Video, Elastic Bands Content: Strength training, balance training, psychological counseling	3 sessions/week × 6 months	Nutrition education	SPPB, AM-PAC, Berg
Yafit Gilboa et al. (2019)	Palestine	RCT	30(remote), 30/30(face-to-face)	Form: Video Conferencing(iPad® devices and Skype™ software) Content: Task-oriented rehabilitation, implementation strategies, and assessment of progress	Weekly × 10 weeks	Community care	FIM, SF-12, GDS, etc.
Sarah Eichler et al. (2017)	Germany	RCT	55/55	Form: Interactive Telerehabilitation System(MyRehab®) Content: Exercise training, video conferencing	3-4 sessions/week × 3 months	Standard physiotherapy	6MWT, TUG, 5STS, WOMAC, Patient adherence, etc.
Mark Ehioghae et al. (2024)	USA	systematic reviews	835 patients	Form: Virtual Reality(Nintendo Wii Fit™, and 3D tracking technology) Content: Functional rehabilitation, pain relief, increase patient engagement and satisfaction	≥12 weeks	Traditional rehab	VAS, WOMAC, Patient satisfaction, etc.
Kui Ching Cheng et al. (2022)	HongKong, China	RCT	19/20	Form:Mobile App(smartphones or tablets) Content: Rehabilitation content, video guidance, patient progress tracking, caregiver skills video library	Daily × 2 months	Booklet training	MFAC, EMS, LEFS, Patient adherence, etc.
Bernardo Abel Cedeno-eloz et al. (2024)	Spanish	RCT	87/87	Form: Mobile App and Multi-component Training (ActiveHip + and Vivifrail) Content: Exercise rehabilitation, nutritional intervention,resistance, balance, flexibility and cardiovascular endurance training	5 sessions/week × 12 months (3 months ActiveHip+ + 9 months Vivifrail)	Multidisciplinary clinic	SPPB, EuroQol-5D, FAC, etc.
Chiara Busso et al. (2020)	Italy	RCT	28/28	Form: Digital Platform(ReHub) Content: Rehabilitation exercise, real-time feedback,data logging	Daily × 3 weeks	Printed guide	TUG, FIM, HOOS, ROM, etc.
Piyapat Dajpratham et al. (2025)	Thailand	RCT	16/17	Form: Line App Real-Time Video Conferencing and Text-Image Video Workbook Content: Resistance training (progressive strengthening of upper and lower limbs)	3 sessions/week × 6 weeks + 2 sessions/week × 6 weeks	Pamphlet	SPPB,2MWT,etc.
Xiangmei Shui et al. (2025)	China	RCT	69/69	Form: WeChat App Content: Rehabilitation exercise, video assessment	1 session/month (45 min/session)	Routine care	FIM,HHS,NRS,HAMA HAMD,etc.

This is the legend for Table 2.

Note: HHS = Harris Hip Score. SAS = Self-Rating Anxiety Scale. SDS = Self-Rating Depression Scale. SF-36 = 36-Item Short Form Health Survey. ADL = Activities of Daily Living. BPOMS = Brief Profile of Mood States. HOOS = Hip Disability and Osteoarthritis Outcome Score. SER = Self-Efficacy for Rehabilitation. EQ-5D = EuroQol five-Dimensions Questionnaire. HADS-A = Hospital Anxiety and Depression Scale-Anxiety. HADS-D = Hospital Anxiety and Depression Scale-Depression. TUG: = Timed Up and Go Test. SPPB = Short Physical Performance Battery. FIM = Functional Independence Measure. 6MWT = 6-Minute Walk Test. 10MWT = 10-Meter Walk Test. 2MWT = Two-minute Walk Test.NPRS = Numeric Pain Rating Scale. NRS = Numeric Rating Scale. AM-PAC = Activity Measure for Post-Acute Care. Berg = Berg Balance Scale. SF-12 = 12-Item Short Form Health Survey. GDS = Geriatric Depression Scale. 5STS = 5-Times Sit-to-Stand Test. WOMAC = Western Ontario and McMaster Universities Osteoarthritis Index. VAS = Visual Analog Scale. MFAC = Modified Functional Ambulation Classification. EMS = Elderly Mobility Scale. LEFS = Lower Extremity Functional Scale. FAC = Functional Ambulation Categories.HAMA = Hamilton Anxiety Scale.HAMD = Hamilton depression scale.

### Forms of implementation of remote rehabilitation in older adults postoperative hip fracture patients

The implementation of remote rehabilitation in older adults postoperative hip fracture patients takes various forms, which can be summarized as follows: (1) WeChat platform: one study [11] applied WeChat live video broadcasting to provide patients with functional exercise, dietary guidance, and multi-personal heart-to-heart communication; 3 studies [9,15,17] investigated the implementation of remote rehabilitation through WeChat applets combined with real-time video guidance to provide a personalized exercise plan and real-time feedback. (2) Mobile application (APP): This is the most mainstream approach, with 10 studies [13,14,16,20–23,25–27] integrating text, images, and video interactions to provide patients with functional exercise guidance, health education, and real-time interaction; (3) Video interactive system: 3 studies [11,12,24] used a video interactive system to provide remote rehabilitation guidance to patients; 1 study [18] used pre-recorded DVD videos as a supplement. (4) Telephonic continuity of care: 2 studies [14,27] provided health education, rehabilitation guidance, and advice on preventing complications through regular telephone follow-ups. (5) Wearable devices: 2 studies [22,23] integrated sensors with cloud platforms to provide personalized rehabilitation plans for total hip replacement patients, enabling real-time monitoring of exercise data and plan adjustments. (6) Virtual reality (VR) devices: 1 study [19] utilizing VR technology to create immersive, multi-sensory environments that provide dynamic, interactive rehabilitation training for postoperative patients.

### Intervention content of telerehabilitation in-home care for older adults postoperative hip fracture patients

The intervention content of telerehabilitation includes rehabilitation program guidance, rehabilitation monitoring and assessment, and psychological support: (1) Rehabilitation Program Guidance: This constitutes the core component. Multiple studies indicate [9,11–14,16–21,23–27] that rehabilitation program guidance primarily delivers personalized functional exercise plans—such as hip flexion/extension training, gait training, and balance training—via mobile apps, live streaming platforms, or video instruction. (2)Rehabilitation Monitoring and Evaluation: Approximately half of the studies [9,16,19,20,22–25,27] employed sensors and digital platforms to monitor patients’ exercise intensity, gait patterns, and pain feedback, enabling healthcare providers to dynamically adjust rehabilitation plans. (3)Psychological Support: Some studies [12,15,18,21]integrated psychological support throughout the intervention process. This approach alleviated patients’ emotional distress through family accompaniment, healthcare professional counseling, or digital technology features, thereby enhancing their confidence in recovery and treatment adherence. Additionally, some studies [14–16] also provide resources, including wound care guidance, dietary advice, medication knowledge, peer support, and a video library on caregiving skills.

### Effectiveness of telerehabilitation on older adults postoperative hip fracture patients

The application effect of telerehabilitation focuses on several aspects, including hip joint function, gait stability and walking ability, quality of life, psychological status, pain status, and exercise adherence. The specific results are as follows:

(1) Hip function:4 studies [11,13,14,17] reported changes in Harris scores, demonstrating that patients in the observation group showed significant improvement in hip function during postoperative follow-up. In Shui et al.‘s study [17], although there was no significant difference in HHS scores between the two groups at discharge (P=0.439), the HHS scores in the observation group were significantly higher than those in the control group at 1 month (P=0.006), 3 months (P<0.001), 6 months (P < 0.001), and 9 months postoperatively, the HHS scores in the observation group were significantly higher than those in the control group.Notably, at 9 months postoperatively, the observation group’s score (91.09 ± 3.99) was significantly higher than that of the control group (87.41 ± 6.77, P < 0.001). Another study [9] reported on HOOS scores, finding that the observation group scored higher than preoperative levels at discharge, 6 weeks post-discharge, and 6 months post-discharge. This suggests that remote rehabilitation interventions have a positive impact on patients’ hip functional mobility. 1 study [23] evaluated the range of motion of the hip (ROM) as an index.
(2) Gait stability and walking ability: 2 studies [22,25] reported significant improvement in the 6 Minute Walk Test (6MWT) observation group.Notably, the findings from Lippi et al.‘s [22] study, which utilized the Step-App® remote monitoring system, were particularly striking, with the intervention group demonstrating significantly greater improvement in 6MWT walking distance compared to the control group (P < 0.0001). The same study also found that the intervention group demonstrated significantly greater improvements in walking speed during the 10-meter walk test (10MWT) (P < 0.0001), indicating the positive impact of remote rehabilitation on walking ability. Another study [26] also demonstrated that the 2-minute walk test (2MWT) results showed the intervention group (38.66 meters) achieved a significantly greater walking distance than the control group (20.41 meters) at 6 weeks, P = 0.032. This suggests that remote rehabilitation intervention can help improve walking ability.

1 study [21] reported that the assessment of the Modified Functional Gait Classification (MFAC), Elderly Mobility Scale (EMS), and Lower Extremity Functional Scale (LEFS) was better than the control group; one study reported that the assessment of the Modified Functional Gait Classification (MFAC), Elderly Mobility Scale (EMS), and Lower Extremity Functional Scale (LEFS) assessments, the experimental group showed a significant increase in EMS and LEFS from baseline to the first month, whereas the control group only showed a rise in LEFS.

(3) Quality of life: 2 studies [12,13] used SF-36 scores to assess patients’ quality of life, with results indicating that the quality of life in the observation group was significantly higher than that in the control group.XU et al. [13]reported that at 6 months post-discharge, the intervention group scored 138.90 ± 5.08, which was significantly higher than the control group’s score of 120.80 ± 5.29 (P < 0.001).In addition, one study [24] used health-related quality of life (HRQoL, as measured by the 12-item short form SF-12) as an indicator of health status. The other study used the SF-36 as an indicator of health status. 2 study [17,21] used the Quality of Life: EuroQol-5D and Sarcopenia and Quality of Life (SarQoL) scale as instruments to measure quality of life.
(4) Psychological status: Regarding psychological status, Niu et al. [14] used the BPOMS scale (lower scores indicate better mood) and found that at 8 weeks post-discharge, the scores in the internet-based continuity of care group (13.36 ± 9.28) were significantly lower than those in the telephone follow-up group (16.87 ± 5.12) (P < 0.0001), indicating superior mood improvement. One study [15] assessed anxiety and depression levels (HADS-A/HADS-D) and found that patients using mobile rehabilitation interventions showed significant improvement in depression levels at 6 weeks post-surgery (P = 0.003), while both anxiety and depression levels were alleviated at 10 weeks post-surgery (P < 0.05). 1 study [12] employed the Self-Rating Anxiety Scale (SAS) and Self-Rating Depression Scale (SDS). The observation group scored significantly lower than the control group on both psychological assessment tools (P < 0.05). Additionally,one study [17] using the Hamilton Anxiety/Depression Scale (HAMA/HAMD) demonstrated that the observation group exhibited significantly lower anxiety and depression scores at 3, 6, and 9 months postoperatively compared to the control group (P < 0.001).
(5) Pain status: Regarding pain management, two studies [17,22] employed the NPRS for assessment. Lippi et al. [22] reported that the intervention group exhibited significantly lower pain intensity at one month postoperatively compared to the control group (P = 0.0421). Shui et al. [17] Similar results were also observed (P < 0.05). Additionally, one study [19] employed VR technology for intervention, demonstrating that VR effectively alleviates pain in patients undergoing orthopedic surgery.
(6) Patient adherence: Regarding exercise adherence, all 4 studies [9,11,16,25] reported superior outcomes in the telerehabilitation group compared to the control group, indicating that telerehabilitation significantly promotes improved exercise adherence among patients.

For example, Cheng et al. [16] found that compliance significantly improved after one month of intervention (P = 0.03). By the second month, compliance in the observation group remained higher than that in the control group; however, no significant difference was observed between the two groups (P = 0.09). Tang et al. [9] reported that this advantage persisted up to 6 weeks and 6 months post-discharge, indicating that remote rehabilitation can effectively promote long-term exercise adherence among patients.

## Discussion

### Telerehabilitation offers diverse intervention modalities for postoperative older adult patients with hip fractures

Findings from this study indicate that telerehabilitation provides significant advantages for the postoperative recovery of older adult patients with hip fractures. It offers diversified interventions using various technical means. These interventions are adaptive and responsive to individual differences.Comparing the effectiveness of different formats revealed a hierarchical pattern of outcomes. Highly interactive formats, such as mobile applications and video systems, produced better results in improving functional metrics (e.g., 6MWT, 10MWT) and long-term exercise adherence. These platforms enable personalized programs, real-time feedback, and progress tracking, all of which are crucial for sustaining engagement. Less interactive formats (e.g., telephone follow-ups) are valuable for providing basic education but are less effective for complex recovery and psychological improvement. This distinction guides clinical selection. Simpler formats, such as WeChat push notifications, are suitable as entry-level options for those with low digital literacy or a preference for passive information consumption. More capable patients benefit more from comprehensive apps or video guidance.

The integration of advanced technologies expands the options for telerehabilitation. A study [22] implemented practices using Step-App®, a novel wearable remote monitoring system on Android smartphones. This app connects to wearable devices and collects real-time exercise data. Healthcare professionals can use this information to monitor rehabilitation progress and adjust intervention plans remotely. The study reported excellent results in functional recovery (as measured by the 6MWT and 10MWT) and pain relief (P < 0.0001).

Additionally, VR-based rehabilitation is being increasingly adopted in postoperative recovery. By creating immersive, feedback-driven training environments, VR enhances the engagement and enjoyment of rehabilitation exercises, offering older adult patients a more proactive and motivating recovery experience. This approach contributes to both pain relief and functional improvement [19].

However, diverse intervention methods bring new challenges. Platform compatibility may vary. Limited digital literacy among patients and restricted access to devices can hinder telerehabilitation. Older adult patients, in particular, may have difficulty operating smart devices. Nursing staff play a vital role in supporting and supervising the rehabilitation process. Their involvement ensures that the implementation and monitoring of rehabilitation plans run smoothly.

Therefore, future efforts to expand telerehabilitation should focus on selecting appropriate intervention strategies based on individual patient conditions and promoting flexible, adaptive rehabilitation models to enhance practical outcomes.

### Telerehabilitation offers rich intervention content but lacks standardized protocols

Current telerehabilitation interventions for older adults recovering from hip fracture surgery show a wide range of content, including functional training, health education, dietary guidance, psychological support, and complication prevention. This diversity highlights the potential of telerehabilitation to meet varied postoperative needs. However, there is no standardized structure or protocol, and studies vary considerably in frequency, intensity, and composition of interventions.

Some studies on functional training emphasize early walking, weight-bearing, progressive resistance, balance, and daily living activities, utilizing remote guidance to support recovery [28]. Still, the best strategies for achieving long-term outcomes remain unclear [29]. Rehabilitation exercises are generally considered safe after various hip surgeries. In one study, patients participated in daily exercise sessions of 20–30 minutes, performing four difficulty levels (e.g., sit-to-stand, hip abduction), with each movement repeated 10 times for two sets [16]. Another study [21]used a structured, smartphone-based program three times a week. Sessions covered resistance, strength, balance, flexibility, and endurance training on alternate days, each lasting 30–60 minutes. Nutritional support, based on GLIM criteria [30], emphasized adequate protein, calcium, and vitamin D to help nutrition and functional recovery [31]. These approaches provide initial evidence to guide standard protocols. Synthesizing existing evidence to develop a flexible framework remains essential. Such a framework should define core training elements, dosage guidelines for frequency, intensity, and duration, as well as principles for adjusting to individual recovery. Flexibility—not a one-size-fits-all model—should be the goal.

Psychological support is inconsistently included in these interventions. Many studies note anxiety and depression after surgery, but only some formally add psychological care—such as assessments and remote counseling—to their protocols. Often, support remains a supplementary rather than central element and is rarely evaluated, making its clinical impact unclear.

Outcome evaluation utilizes a range of tools across studies, including the Harris Hip Score, the HOOS, the six-minute walk test (6MWT), and pain scales. These offer varied insights, but without unified outcome measures, comparing results or standardizing findings is difficult. Future work should identify a core set—such as HHS (function), 6MWT/10MWT (activity), EQ-5D (quality of life), NPRS (pain), and SAS/SDA (psychological)—to improve comparability.

In summary, current telerehabilitation interventions offer comprehensive, evidence-based, and feasible content. However, the lack of standardized protocols complicates both implementation and evaluation. Future research should establish consistent frameworks for intervention structure, session frequency, and assessment measures to ensure effective implementation. Developing systematic care pathways and unified telerehabilitation guidelines is key to improving clinical practice.

### Telerehabilitation demonstrates high feasibility and positive clinical outcomes

Current research broadly supports the high feasibility of telerehabilitation for older adult patients recovering from hip fracture surgery. Numerous studies report favorable clinical outcomes across multiple dimensions. Most interventions rely on commonly available technologies such as smartphones and tablets. Even among older patients, basic telerehabilitation tasks can generally be completed with minimal training. This is especially true when assisted by healthcare providers or family members. Telerehabilitation significantly reduces the burden of travel to and from healthcare facilities, as well as lowering outpatient care costs. It is particularly beneficial for patients living in remote areas or with limited mobility. This convenience enhances rehabilitation outcomes and helps alleviate pressure on healthcare systems [27]. Caregivers of older adults with hip fractures can feel burdened by anxiety, depression, and low back pain. Older patients rely heavily on their caregivers for both physical and psychological support [32–34]. Crotty et al. [35] found that patients recovered at home with similar outcomes to the hospital setting and with reduced caregiver burden. Moreno et al. [36] found that the mHealth intervention improved objective physical functioning and anxiety in older adults with hip fractures. This intervention also benefited family caregiver burden and depression.

Clinically, telerehabilitation has shown positive effects on key functional indicators. Studies [13,22] have reported significant improvements in postoperative Harris Hip Scores, six-minute walk tests (6MWT), and 30-second sit-to-stand tests (30STS). These interventions have also been associated with reduced pain intensity, improved quality of life, and, in some cases, better emotional well-being.

One study demonstrated that a telerehabilitation program for older adult patients with hip fractures helped participants regain up to 96.8% of their pre-fracture Functional Independence Measure (FIM) scores [20]. As a core metric for functional recovery, the FIM [37] evaluates six domains of daily functioning: self-care, sphincter control, mobility, locomotion, communication, and cognition. Higher scores indicate better functional independence. This study also highlighted the proactive role of nurses in the rehabilitation process, with many viewing remote programs as valuable tools for enhancing patients’ self-management capabilities and promoting functional recovery [32].

Additionally, differences in intervention formats significantly impact patient engagement and the quality of training. A study [16] found that rehabilitation guidance via mobile applications improved patient adherence and self-efficacy compared to text-and-image-based materials. Two mechanisms drive this improvement, which paper-based materials cannot replicate. First, video-based instruction breaks down complex movements into manageable modules, utilizing close-up shots and voice prompts to facilitate imitability. This significantly reduces execution errors caused by cognitive limitations or pain interference in older adults. Second, the platform’s automatic reminders and progress tracking help break the “I’ll do it tomorrow” procrastination cycle. Rhythmic prompts and visual achievements maintain behavioral inertia even without face-to-face supervision. The mobile app further integrates rehabilitation tasks into everyday digital life scenarios, such as WeChat messages and phone unlock screens. This combines exercise with existing technological habits to create natural triggers that are easy to understand and remember. Thus, adherence is solidified.

However, the feasibility of telerehabilitation is not universally guaranteed. Challenges such as limited cognitive capacity, inadequate home support, and lack of access to digital devices may hinder some patients from completing interventions as planned.Before recommending telerehabilitation, clinicians should routinely assess patients’ device accessibility, internet connectivity, basic operational capabilities, and the level of family support available. For those with significant barriers, provide alternative solutions or enhanced support. Ongoing optimization is necessary to ensure the accuracy of remote assessments and the safety of interventions.

### Telerehabilitation faces persistent technological barriers to implementation

Despite its proven benefits and operational viability, telerehabilitation for older adults with hip fractures faces notable technological barriers. Our review identifies several main obstacles: (1) Digital Access Divide: Reliable internet connectivity is lacking, especially in rural or underserved regions [38], and some older adults do not have the necessary hardware due to cost or availability. (2) Digital Literacy Gap: Many older adults struggle with navigating complex interfaces, understanding app functions, and troubleshooting. Cognitive decline or limited technological experience can exacerbate these problems, leading to frustration and non-adherence [39,40]. (3) Usability and Design Limitations: Applications not built for older adults may contain small fonts, complicated menus, or inadequate language support. (4) Technical Reliability: Unstable connections, software glitches, and sensor errors reduce trust in the system.

These challenges disproportionately affect vulnerable older adults, emphasizing the need for equitable telerehabilitation.Thus, targeted solutions are vital for clinical adoption. First, select age-friendly devices with clear interfaces, intuitive operation, and voice assistance. Second, provide thorough technical training to patients and caregivers before interventions start. Maintaining accurate remote assessments and ensuring the safety of interventions are essential for future system advancements and clinical protocols.

## Conclusion

Telerehabilitation for older adults after hip fracture surgery improves functional recovery and treatment adherence. Its flexible formats and promising clinical outcomes depend on matching intervention types to patients’ digital literacy. Effective implementation requires standardizing rehabilitation modules and outcome measures. Despite the positive results, barriers such as operational difficulties and resource shortages persist. Integrating telerehabilitation into follow-up care, guided by healthcare professionals, will enhance effectiveness and scalability. Ongoing focus should be placed on standardized pathways and individualized strategies.

## Supporting information

S1 Fig Flowchart of literature screening. (TIF)

S2 Files PRISMA-ScR Checklist. (PDF)
